# Associations between Low to Moderate Consumption of Alcoholic Beverage Types and Health Outcomes: A Systematic Review

**DOI:** 10.1093/alcalc/agab082

**Published:** 2021-12-11

**Authors:** Ramon Estruch, Henk F J Hendriks

**Affiliations:** Department of Internal Medicine, Hospital Clinic, Institut d’Investigacions Biomèdiques August Pi i Sunyer (IDIBAPS), University of Barcelona, Villarroel, 170, 08036 Barcelona, Spain; CIBER Fisiopatología de la Obesidad y la Nutrición (CIBER OBN), Instituto de Salud Carlos III, Montforte de Lemos 3-5, Pabellón 11, Planta O / 28029 Madrid, Spain; Hendriks Nutrition Support for Business, Laan van Cattenbroeck 70, 3703 BP Zeist, The Netherlands

## Abstract

**Aims:**

There is limited research comparing light to moderate wine, beer and spirits consumption and their impact on long-term health. This systematic review aims to investigate the studies published in the past 10 years and qualitatively assess the similarities and differences between the three main beverages, when consumed at a low to moderate level, for their associations with various health outcomes.

**Methods:**

A systematic search was conducted for comparative studies published in English language (2010 to mid-2021) of beverage-specific low to moderate alcohol consumption associated with all-cause mortality, cancer, cardiovascular disease and diabetes mellitus type II.

**Results:**

The search yielded a total of 24 studies (8 meta-analyses; 15 prospective studies and 1 pooled analysis). Overall, most studies showed similar associations of different alcoholic beverages with chronic conditions, including all-cause mortality, many types of cancer, cardiovascular disease and diabetes mellitus type II. Not all data are consistent. Some studies show more beneficial or detrimental effects of wine than other beverage types, whereas other studies show such effects for other beverages.

**Conclusion:**

Moderate consumption of one specific alcoholic beverage (wine, beer or spirits) may not be consistently associated with higher or lower risks for common health outcomes as compared with moderate consumption of any of the other alcoholic beverages.

## Teaser

**Short Summary:** A systematic review of 24 prospective studies and meta-analyses showed that moderate consumption of one specific alcoholic beverage (wine, beer or spirits) may not be consistently associated with higher or lower risks for common health outcomes as compared with moderate consumption of any of the other alcoholic beverages.

## INTRODUCTION

Short-term harmful effects related to alcohol abuse are most often the result of excessive consumption and binge drinking and include acute alcohol intoxication, injuries and violence (Centers for Disease Control and Prevention, 2021). Long-term excessive drinking and binge drinking also result in many negative health effects. However, short-term and long-term light to moderate alcohol consumption have been associated with several positive health outcomes (Hendriks, 2020). Consequently, many countries have guidelines advising low to moderate alcohol consumption aimed at reducing the health burden caused by the misuse of alcohol (WHO, 2018).

Most large-scale prospective studies and meta-analyses (MAs) investigating the relationships between alcohol consumption and health outcomes have assumed that health outcomes are linked to the level of alcohol exposure, regardless of the underlying beverage type (Rehm and Hasan, 2020). The WHO and the UN do not differentiate alcoholic beverage types when discussing alcohol related topics (WHO, 2018). The ‘standard drink’ adopted by governments and drinking guidelines in many countries are defined by ethanol content alone (Haseeb *et al*., 2017). Furthermore, the legal liability from being under the influence of alcohol does not distinguish between beverage types, either.

However, some studies have reported that non-ethanol content, like polyphenols abundant in red wine or vitamins and minerals in beer, may exert additional beneficial effects on health (Van Der Gaag *et al*., 2000; Estruch *et al*., 2004). Distilled spirits are widely regarded as more harmful than wine and beer, even at equivalent levels of consumption. Consequently, there is a perception that both the detrimental as well as the beneficial health aspects associated with the consumption of alcoholic beverages are associated with specific alcoholic beverages (Room *et al*., 2011).

Limited research summarizes evidence for the associations of low to moderate drinking of the three beverage types and the most common diseases, specifically cancer, cardiovascular disease, diabetes mellitus type II and the overall outcome, all-cause mortality. This systematic review summarizes scientific studies on these associations, specifically prospective studies and MAs published in the past 10 years.

## MATERIALS AND METHODS

### Search strategy

The electronic databases PubMed, Embase and Web of Science were searched for inclusion of full-length articles, written in English roughly between January 2011 and March 2021 to assess the latest relevant scientific evidence available. The key words included (all fields): ‘(wine or wines) and (beer or beers) and (spirits or spirits) and (human)’. The search strategy for all three databases is provided in the Supplementary Materials. In addition, additional relevant papers from the period January 2010 through July 2021 were identified by reviewing reference lists in retrieved articles and additional searches in electronic databases.

### Study eligibility

All titles and abstracts were screened to include eligible studies. Full-text articles were assessed for further eligibility. Articles concerning non-human studies were excluded. Human studies had to meet the following criteria: (a) the study had a prospective design or the study was a MA of prospective studies mainly (>50%); (b) the outcomes were: all-cause mortality, any type of cancer incidence and/or mortality, cardiovascular disease incidence and/or mortality and diabetes mellitus type II incidence and/or mortality; (c) the study reported relative risk (RR), hazard ratio (HR) or odds ratio (OR) and its 95% confidence intervals for drinking beer, drinking wine and drinking spirits; and (d) the study had to report on at least three drinking levels including a control group, which either consisting of a non-drinking group or an occasional/light drinking group. Alcohol consumption in control groups varied between 0 gram per day for teetotallers or current non-drinkers or an occasional drinker ranging up to a maximum of 10-gram alcohol/day in one study (Jensen *et al*., 2012). One other alcohol consumption group at least consisted of a light to moderate drinking group. The light to moderate drinking group closest to the American dietary guidelines for moderate alcohol consumption, viz. 28-gram alcohol/day for men and 14-gram alcohol/day for women, was chosen for comparison to the control group only. These quantities for both the control group and light to moderate drinking group are specified in Table 2. Open-end groups, even when defined as ‘1+ drinks a day’ were generally not used for comparisons, except for the spirits drinking women in one study (Naudin *et al*., 2018). This systematic review was conducted according to preferred reporting items for systematic reviews and meta-analyses (PRISMA) guidelines.

**Fig. 1:**
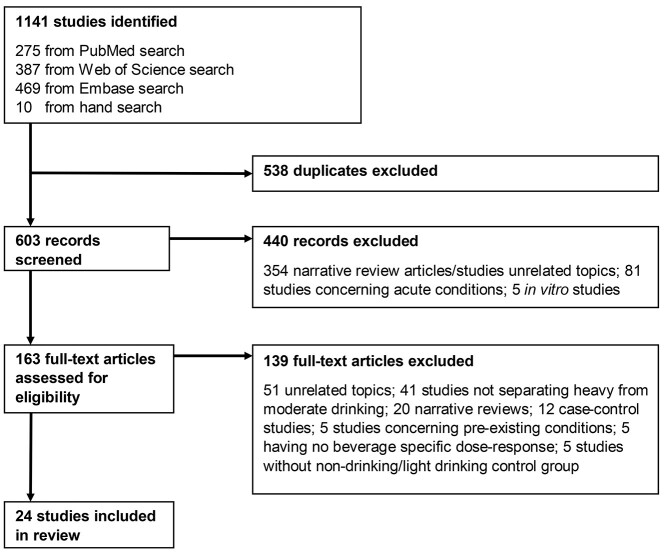
PRISMA flow diagram of systematic study review.

### Data extraction

Study screening and selection was prepared by two people (ZF and NI) and reviewed by the authors (HH and RE). The risk of bias was negated by independently screening and selection of the search results in separate documents and later identifying discrepancies between the two reviewers (ZF and NI) and by utilizing a checklist pre-determined before the final selection was executed (HH and RE).

For each selected study, descriptive information was extracted including the type of study (prospective, MA or pooled analysis [PA]), probability expression, region, observation period, number of cases, person years and adverse event reporting endpoint (Table 1). Table 2 summarizes endpoint, alcohol consumption registration, control group and alcohol consumption group used for comparison and the risks associated with light to moderate consumption of wine, beer or spirits extracted from the selected papers. The measure of association (e.g. RR) and a measure of statistical uncertainty of the measure of association (confidence interval) were also specified. When risks were not reported in tables, figures depicting dose–response relations for all three alcoholic beverages and risks for disease outcome were used to extract the measures of association (Xu *et al*., 2015; Huang *et al*., 2017; Zhou *et al*., 2017; Sun *et al*., 2020). Risks were given in bold when risks were significant, namely different from 1; bold non-italics for increased risks and bold italics for a decreased risk per alcoholic beverage. Differences in risk associations between with the various alcoholic beverages are described in the text.

**Table 1:** Summary of the characteristics of 24 studies included

#	First author (year)	Study type (MA/ CS/PA)	RR/HR/ OR	Region/Country	Observation period (year)	Population size (×10^3^)	Number of cases (×10^3^)	Person years^a^ (×10^6^)	I or M	Endpoint
1	Di Castelnuovo (2021)	CS	HR	Europe	11.8	143	17	1.7	M	All-cause mortality
2	Kim (2019)	MA	RR	World	8	31	8.4	0.25	M	All-cause mortality
3	Sun (2020)	MA	RR	World	NS	NS	45.3	NS	I	Breast cancer
4	Hong (2020)	MA	RR	World	NS	NS	21^b^	NS	I	Aggressive and non-aggressive prostate cancer
5	Chao (2011)	CS	HR	USA	6	66	0.6	0.4	I	Lung cancer
6	Park (2019)	CS	HR	USA	16.7	191	5	3.2	I	Colorectal cancer
	Kim (2019)	MA	RR	World	9	22	4.7	0.2	M	Colorectal cancer
7	Rivera (2016)	MA	HR	USA	18	210	1.3	3.8	I	Skin melanoma
8	Jensen (2012)	CS	HR	Denmark	11.4	55	2.6	0.6	I	Basal and squamous cell carcinoma
9	Botteri (2017)	CS	HR	Europe	14	476	1.8	6.6	I	Non-aggressive urothelial cell carcinoma
10	Lew (2011)	CS	RR	USA	9	492	1.8	4.4	I	Renal cell carcinoma
11	Xu (2015)	MA	RR	World	8.6	4867	5.5	410	I & M	Renal cell carcinoma
12	Karami (2015)	CS	HR	USA	11.4	108	0.4	1.2	I	Renal cell carcinoma
13	Zhou (2017)	MA	RR	World	13.4	1600	9.8	21.4	I	Endometrial cancer
14	Genkinger (2006)	PA	RR	World	8.7	530	2	4.6	I	Ovarian cancer
15	Duell (2011)	CS	HR	Europe	8.7	478	0.4	4.2	I	Gastric cancer
16	Wang (2018)	CS	HR	USA	13.5	490	1.4	6.6	I	Gastric cancer
17	Gapstur (2011)	CS	RR	USA	24	1030	6.8	24.7	M	Pancreatic cancer
18	Wang (2016)	MA	RR	World	11.7	4200	11.8	49.1	I	Pancreatic cancer
19	Naudin (2018)	CS	HR	Europe	14	476	1.3	6.7	I	Pancreatic cancer
20	Song (2018)	CS	HR	USA	2.9	156	6.2	0.5	I	Coronary artery disease
	Di Castelnuovo (2021)	CS	HR	Europe	11.8	143	5.5	1.7	M	Cardiovascular disease
21	Holst (2017)	CS	HR	Denmark	4.9	71	1.7	0.3	I	Diabetes type II
22	Marques-Vidal (2015)	CS	OR	Suisse	5.5	5	0.3	0.03	I	Diabetes type II
23	Huang (2017)	MA	RR	World	9.3	497	20.6	4.6	I	Diabetes type II
24	Cullman (2012)	CS	OR	Sweden	9	5	0.2	0.05	I	Diabetes type II

CS: cohort study; I or M: incidence or mortality; NS: not specified.

^a^Person years in millions: observation period x population size.

^b^Concerning up to 3000 cases for aggressive prostate cancer and up to 18,000 cases for non-aggressive prostate cancer.

**Table 2:** Associations between light to moderate beer, wine and spirits consumption and health outcomes reviewed

#	First author (year)	Endpoint	Alc. cons. Registration	Control group	Alc. cons. Category	Ratio (confidence interval)		
						Wine consumption	Beer consumption	Spirits consumption
1	Di Castelnuovo (2021)	All-cause mortality	Unclear	Life-time abstainer	0.1–10 g/day	***0.87 (0.81–0.93)***	0.96 (0.90–1.03)	0.94 (0.88–1.00)
			Unclear	Life-time abstainer	10–20 g/day	***0.81 (0.71–0.91)***	**1.20 (1.06–1.34)**	**1.15 (1.03–1.29)**
2	Kim (2019)	All-cause mortality	Unclear	Non-drinker	<12.5 g/day	***0.85 (0.75–0.96)***	0.94 (0.80–1.10)	0.95 (0.86–1.06)
3	Sun (2020)	Breast cancer	Current	Non-drinker	14 g/day	**1.20 (1.10–1.30)**	1.05 (0.87–1.25)	1.12 (0.95–1.37)
4	Hong (2020)	Non-aggressive Prostate cancer	Unclear	Non-drinker	28 g/day	**1.05 (1.01–1.09)**	**1.05 (1.01–1.08)**	**1.07 (1.04–1.11)**
		Aggressive prostate cancer	Unclear	Non-drinker	28 g/day	1.07 (0.97–1.17)	***0.79 (0.70–0.90)***	1.02 (0.95–1.11)
5	Chao (2011)	Lung cancer	Baseline	<once/month	<12 g/day	0.97 (0.79–1.18)	1.08 (0.85–1.37)	0.89 (0.71–1.12)
6	Park (2019)	Colorectal cancer	Current	Never/hardly ever	5–14 g/day	0.94 (0.68–1.31)	1.06 (0.87–1.31)	1.14 (0.76–1.71)
	Kim (2019)	Colorectal cancer	Unclear	Non-drinker	< 12.5 g/day	***0.82 (0.69–0.97)***	1.03 (0.90–1.18)	0.90 (0.80–1.01)
7	Rivera (2016)	Skin melanoma	Lifetime	0 - < 12 g/month	3.5–7 g/day	1.12 (0.68–1.85)	0.96 (0.72–1.28)	1.01 (0.78–1.31)
8	Jensen (2012)	Basal and squamous cell carcinoma	Current	0 - < 10 g/day	10–30 g/day	**1.14 (1.01–1.29)**	**1.18 (1.05–1.34)**	1.06 (0.76–1.48)
9	Botteri (2017)	Non-Aggressive urothelial cell carcinoma in men	Lifetime	0–6 g/day	12–24 g/day	0.91 (0.72–1.15)	1.04 (0.83–1.31)	1.04 (0.80–1.36)
		Non-aggressive Urothelial cell carcinoma in women	Lifetime	0–3 g/day	3–12 g/day	0.90 (0.69–1.16)	0.96 (0.63–1.47)	1.22 (0.90–1.65)
10	Lew (2011)	Renal cell carcinoma men	Current	0–5 g/day	5–15 g/day	0.95 (0.77–1.17)	***0.81 (0.66–0.99)***	0.84 (0.64–1.10)
		Renal cell carcinoma women	Current	0–5 g/day	5–15 g/day^a^	0.95 (0.50–1.82)	0.78 (0.55–1.12)	0.85 (0.56–1.29)
11	Xu (2015)	Renal cell carcinoma	Unclear	Non-drinker	14 g/day	***0.83 (0.72–0.97)***	***0.68 (0.55–0.80)***	***0.88 (0.80–0.97)***
12	Karami (2015)	Renal cell carcinoma	Current	Non-drinker	2–10 g/day	0.93 (0.65–1.32)	0.92 (0.68–1.24)	0.93 (0.65–1.32)
13	Zhou (2017)	Endometrial cancer	Unclear	Non-drinker	14 g/day	0.96 (0.88–1.12)	1.00 (0.93–1.06)	0.86 (0.66–1.19)
14	Genkinger (2006)	Ovarian cancer	Baseline	Non-drinker	5–14 g/day	1.03 (0.86–1.23)	0.75 (0.56–1.01)	1.02 (0.75–1.38)
15	Duell (2011)	Gastric cancer	Baseline	0.1–4.9 g/day	5–30 g/day^b^	1.13 (0.83–1.54)	1.10 (0.74–1.64)	1.08 (0.71–1.63)
16	Wang (2018)	Gastric cardia adenocarcinoma	Current	Non-drinker	14 g/day	0.95 (0.78–1.15)	0.91 (0.74–1.11)	0.98 (0.80–1.20)
		Gastric non-cardia adenocarcinoma	Current	Non-drinker	14 g/day	0.88 (0.73–1.06)	1.03 (0.85–1.25)	0.89 (0.74–1.08)
17	Gapstur (2011)	Pancreatic cancer	Baseline	Non-drinker	24 g/day	0.91 (0.68–1.20)	1.08 (0.86–1.35)	1.15 (0.98–1.35)
18	Wang (2016)	Pancreatic cancer	Unclear	Low or non-drinking	12–24 g/day	0.95 (0.85–1.07)	1.05 (0.93–1.19)	1.09 (0.99–1.19)
19	Naudin (2018)	Pancreatic cancer men	Baseline	0.1–2.9 g/day	10–20 g/day^c^	1.03 (0.82–1.29)	1.02 (0.80–1.29)	0.93 (0.64–1.35)
		Pancreatic cancer women	Baseline	0.1–2.9 g/day	3–10 g/day^d^	0.98 (0.80–1.20)	0.87 (0.65–1.16)	**1.45 (1.09–1.94)**
20	Song (2018)	Coronary artery disease	Current	Non-drinker	6–14/28 g/day	***0.83 (0.73–0.94)***	***0.74 (0.65–0.84)***	***0.76 (0.67–0.87)***
	Di Castelnuovo (2021)	Cardiovascular disease	Unclear	Life-time abstainer	0.1–10 g/day	***0.84 (0.74–0.96)***	0.98 (0.87–1.11)	0.98 (0.87–1.09)
			Unclear	Life-time abstainer	10–20 g/day	0.84 (0.66–1.05)	1.14 (0.92–1.41)	0.98 (0.80–1.19)
21	Holst (2017)	Diabetes type II	Baseline	< 2 g/day	<10.5 g/day	0.88 (0.70–1.10)	***0.70 (0.56–0.87)***	1.23 (0.93–1.63)
22	Marques-Vidal (2015)	Diabetes type II	Current	Non-drinker	<18 g/day	0.64 (0.38–1.08)	1.00 (0.71–1.40)	1.02 (0.72–1.44)
23	Huang (2017)	Diabetes type II	Unclear	Non-drinker	14 g/day	***0.82 (0.76–0.89)***	0.93 (0.84–1.00)	0.96 (0.86–1.07)
24	Cullman (2012)	Diabetes type II	Baseline	<1 g/day	1–5 g/day	0.49(0.21–1.15)	1.28 (0.63–2.62)	***0.39 (0.17–0.91)***

Ratios in bold non-italics represent increased ratios for an alcoholic beverage. Ratios in bold-italics represent decreased ratios for an alcoholic beverage.

^a^Concerning the 5^+^ g/day category.

^b^Beer and wine: 10–30 g/day and spirits: 5–10 g/day.

^c^For spirits 5–10 g/day.

^d^For spirits 2–5 g/day.

## RESULTS

### Literature search

Of the 1141 studies initially identified, 538 duplicates were excluded. Of the 603 remaining articles title and abstract were screened yielding 163 articles published between 2011 and 2021 concerning beer, wine and spirits consumption possibly related to the health outcomes of choice. Of these 163 articles the full text was browsed, excluding 50 studies unrelated to the health outcomes of choice, 41 studies not separating heavy from moderate drinking, 20 narrative reviews, 12 case-control studies, 5 studies concerning pre-existing conditions, 5 not having beverage-specific dose responses and 5 studies without a non-drinking/light drinking control group. This selection process therefore excluded 139 studies resulting in 24 studies to be included in our systematic review (Figure 1).

Study characteristics specified in Table 1 include study type, probability expression, region, observation period, number of cases, person years and adverse event endpoint. All of the 24 included studies were prospective cohort studies or MAs and one PA (Genkinge 2006). Two studies of these 24 reported on all-cause mortality and one additional disease, namely cardiovascular disease (Di Castelnuovo *et al*., 2021) and colorectal cancer (Kim *et al*., 2019), 18 concerned a specific type of cancer, only 2 prospective studies concerned cardiovascular disease and 4 articles studied beverage-specific association with diabetes mellitus type II incidence.

### All-cause mortality

Two studies on all-cause mortality were selected (Table 2); one prospective study (Di Castelnuovo *et al*., 2021) and one MA (Kim *et al*., 2019). The prospective study showed a risk reduction associated with wine consumption and a borderline significant risk reduction for the other beverages at light drinking levels (i.e. 0.1–10 g/day). At higher consumption levels (i.e. 10–20 g/day) a risk increase was associated with all-cause mortality for those consuming beer and those consuming spirits but a risk decrease for those consuming wine. The MA (Kim *et al*., 2019) also showed a risk reduction for those consuming wine, but no risk increase for those drinking beer or spirits.

### Studies on cancer

Eighteen studies on cancer were identified covering the most common cancer types in men and women (breast, prostate, lung, colorectal, bladder and melanoma) as well as some additional less common cancers (Table 2). Cancers known to be associated with alcohol consumption, namely breast cancer (Sun *et al*., 2020) and colorectal cancer (Kim *et al*., 2019; Park *et al*., 2019) were reported on. The MA on breast cancer showed an increase in breast cancer incidence; however, the increase was only significant for women consuming wine. Studies on colorectal cancer included MA on colorectal cancer mortality (Kim *et al*., 2019) and a prospective study on colorectal cancer incidence (Park *et al*., 2019). Both studies showed non-significant associations with colorectal cancer incidence with the exception of a decrease in colorectal cancer incidence in those consuming <12.5 gram alcohol from wine per day.

Some other cancers known to be associated with moderate to light alcohol consumption, such as cancers of the oral cavity, pharynx, larynx, oesophagus and liver, were not reported on during this time period in a way that allowed selection for this review.

An additional 15 articles reported on cancers that may or may not be associated with light to moderate wine, beer and spirits consumption. These included non-aggressive and aggressive prostate cancer and cancers of the lung, skin, bladder, kidney, endometrium, ovary, stomach and pancreas (Table 2). Seven of these studies did not show differences in the association between beverage-specific consumption and cancer incidence or mortality. These include a prospective study on lung cancer incidence (Chao *et al*., 2011), a prospective study on bladder cancer incidence in men and women (Botteri *et al*., 2017), MA on endometrial cancer incidence (Zhou *et al*., 2017) and PA on ovarian cancer incidence (Genkinger *et al*., 2006). MA on skin melanoma incidence (Rivera *et al*., 2016) did not show an association with any specific alcoholic beverage consumed in moderation. Another, prospective study on non-melanoma, basal and squamous cell carcinoma incidence (Jensen *et al*., 2012), however, showed positive associations for those consuming wine and beer, but not for those consuming spirits. One prospective study on gastric cancer incidence (Duell *et al*., 2011) did not show an association with moderate alcohol consumption nor a difference in risk for wine, beer and spirits consumers. MA (Wang *et al*., 2018) on gastric cancer showed no beverage-specific changes in risk.

Three selected studies concerned renal cell carcinoma. A prospective study (Lew *et al*., 2011) reported separately on men and women showing non-significant risk reductions with a significant reduction in beer consuming men only. Similar results, without significant risk reductions in one specific drinking group were observed in a more recent prospective study (Karami *et al*., 2015). A large MA also showed no differences in risk reductions between the three beverages, but risk reductions were all significant for all three alcoholic beverages (Xu *et al*., 2015).

One prospective study on pancreatic cancer incidence (Naudin *et al*., 2018) showed an increase in risk for female spirits consumers, but not for male spirits consumers. This outcome was not confirmed by a second prospective study on pancreatic cancer mortality (Gapstur *et al*., 2011), nor by MA on pancreatic cancer incidence (Wang *et al*., 2016) showing no association of moderate alcohol consumption with pancreatic cancer incidence.

MA on prostate cancer (Hong *et al*., 2020) showed an increased risk for non-aggressive prostate cancer with no differences between consuming one of the three beverages and a risk reduction for aggressive prostate cancer incidence associated with beer consumers only.

Of the 18 selected studies on alcohol consumption and cancer, no beverage-specific risk increase appeared to occur in 15 studies. Two studies showed a risk increase for one beverage only: for wine (Sun *et al*., 2020) and for spirits consumed by women (Naudin *et al*., 2018). Only one study showed a risk increase for two beverages, i.g. wine and beer (Jensen *et al*., 2012). Three studies showed a risk reduction for one beverage only: for wine (Kim *et al*., 2019) and for beer (Lew *et al*., 2011; Hong *et al*., 2020).

### Studies on coronary artery disease

Only two prospective studies on cardiovascular disease associated with the consumption of wine, beer and spirits were selected, one prospective study on coronary artery disease incidence (Song *et al*., 2018) and one multicentre prospective study on cardiovascular disease mortality (Di Castelnuovo *et al*., 2021). Risk reductions were observed for all three beverages in the American study; the European study found a significant risk reduction for wine drinking only.

### Studies on diabetes mellitus type II

Four studies on the association between consuming a specific beverage and risk for type II diabetes were selected, of which three were prospective studies and one was MA. The prospective studies were all relatively small with varying results; one reported a risk reduction for spirits consumption only (Cullmann *et al*., 2012), another reported a risk reduction for beer consumption only (Holst *et al*., 2017) and one reported no significant risk changes associated with any of the beverages. The MA (Huang *et al*., 2017) showed risk reductions being significantly associated with wine consumption only.

## DISCUSSION

This systematic review based on 24 papers reporting on several main health outcomes associated with the consumption of light to moderate wine, beer or spirits suggests that moderate consumption of one specific alcoholic beverage would not be consistently associated with higher or lower risks for major health outcomes as compared with moderate consumption of any of the other alcoholic beverages.

It has been debated for a long time whether moderate consumption of either wine, beer or spirits would have greater health benefits or alternatively whether alcohol abuse of one of these three beverages would have a greater negative health impact (Rehm and Hasan, 2020). Difficulties to assess potential differences in health outcomes associated with the consumption of one specific alcoholic beverage are many. Confounding may occur in various aspects, like cultural aspects. However, there seems to be no general pattern regarding the relationship between beverage choice and health or harm outcomes, which holds across cultures (Mäkelä *et al*., 2011). Moreover, socio-demographic and economic factors show an overall major impact on consumption changes (Allamani *et al*., 2014) rather than beverage type consumption.

In addition, a consumer preferring wine may differ from a consumer preferring beer, not only in drinking pattern, but also in background diet (Johansen *et al*., 2006) and other life-style factors. Drinking pattern is relevant, not only in terms of quantity, but also in terms of frequency (Haseeb *et al*., 2017). Alcoholic beverages may be combined with a meal, generally yielding lower blood alcohol concentrations than alcoholic beverages consumed without a meal. Drinking alcohol regularly and in moderation with a meal in combination with other healthy life style factors may be associated with the best possible health outcome (Mukamal *et al*., 2006). Also, currently, many consumers do not drink one type of beverage only. All in all this suggests that prospective studies may not be optimally suited to correct for all confounding factors disturbing the association of interest. However, use of the available studies on light to moderate alcohol consumption reporting beverage-specific consumption in a dose-dependent way may add to the current body of evidence.

The two studies on all-cause mortality (Kim *et al*., 2019; Di Castelnuovo *et al*., 2021) show a small risk reduction associated with light (<12.5 g alcohol/day) drinking consistent with older studies on the topic both in prospective studies (Thun *et al*., 1997) and reviews (Jayasekara *et al*., 2014). The resulting J-shaped curve describing the association between alcohol drinking and all-cause mortality has been criticized for many decades. Main criticism is that the control group, when consisting of teetotallers, may have included persons that have had a history of alcohol-abuse. This criticism has led to a re-evaluation of the data and after excluding former drinkers the association between alcohol consumption and all-cause mortality remained essentially the same (Di Castelnuovo *et al*., 2006). A similar analysis was performed in the prospective study selected for this review (Di Castelnuovo *et al*., 2021) with again essentially similar results. The studies selected for this review used various control groups such as current non-drinkers, lifetime abstainers and occasional drinkers.

In both the prospective study (Di Castelnuovo *et al*., 2021) and the MA, concerning <1 million person years (Kim *et al*., 2019) wine appeared to be more beneficial as compared with beer and spirits. One possible explanation may be that other factors such as lifestyle and specifically the diet, such as the Mediterranean diet, may contribute to that effect. The Mediterranean diet includes moderate consumption of alcohol—in particular, red wine—always with meals. Also, the Mediterranean diet includes high intake of olive oil, fruit, nuts, vegetables and cereals; a moderate intake of fish and poultry; a low intake of dairy products, red meat, processed meats and sweets. Several studies have demonstrated that adhering to the Mediterranean diet is beneficial for cardiovascular health (Estruch *et al*., 2018) and all-cause mortality (Gea *et al*., 2014). Therefore, benefit of wine consumption may be prone to confounding by diet. In fact, it has been observed that wine drinkers tend to have a healthier diet and behaviour (Johansen *et al*., 2006).

The majority of studies selected on cancer risk showed similar outcomes for wine, beer and spirits. Alcoholic beverages and ethanol have been categorized as carcinogenic to humans (Group 1; IARC Working Group on the Evaluation of Carcinogenic Risks to Humans, 2010) irrespective of the level of alcohol consumption. Some studies selected in this review suggest that light to moderate alcohol consumption may not be associated with risk for several cancers. These cancers include cancer of the lung, colon-rectum, skin (melanoma), bladder, kidney, endometrium, ovary, stomach and pancreas. However, some of these studies (Chao *et al*., 2011; Jensen *et al*., 2012; Kim *et al*., 2019) concerned <1 million person years and may be underpowered to draw such a conclusion. Some other cancers included in this review, however, may be associated with risk increase at moderate drinking dosages. These include breast cancer (but only for wine consumption), non-aggressive prostate cancer (for all beverages), non-melanoma skin cancer (for wine and beer consumption) and pancreatic cancer (for spirits consuming women only). Yet other cancers were associated with a lower risk; aggressive prostate cancer (for beer consumers only), renal cell carcinoma (in beer drinking men only (Lew *et al*., 2011) and for all beverages (Xu *et al*., 2015). All and all, these outcomes corresponds with the limited contribution of moderate alcohol consumption as a lifestyle factor to overall cancer risk (Hendriks and Calame, 2018).

Two prospective studies on cardiovascular disease met the selection criteria for this review. Both show a reduced risk associated with light to moderate drinking, one for wine mainly (Di Castelnuovo *et al*., 2021) at dosages of 0.1–10 g/day, the other for all three beverages (Song *et al*., 2018) at dosages of 6–14 or 28 g/day. This latter study, however, is small (<1 million person years), but is in agreement with a large MA (Ronksley *et al*., 2011) on alcoholic beverages in general showing that the lowest risk of coronary heart disease mortality occurred with 1–2 drinks (14–28 g alcohol) a day, but for stroke mortality it occurred with ≤1 drink per day. Further substantiation of the beneficial effect of moderate alcohol consumption on cardiovascular diseases comes from nutrition intervention studies. Such studies not only point to the mechanism of protection (Brien *et al*., 2011), but also indicate that the mechanism is very similar when consuming wine, beer or spirits. Main effect of moderate alcohol consumption appears to be the inhibition of the atherosclerotic process (Hendriks, 2020).

Possibly, polyphenols, contained in several alcoholic beverages but mainly in red wine, may also exert protective effects on the cardiovascular system. Polyphenols are not exclusive to wine, but are also present in other alcoholic beverages (i.e. dark beer and malt whiskey), as well as fruit, vegetables, tea and cacao. There is evidence suggesting that ethanol and polyphenols within wine can synergistically confer benefits against chronic cardiovascular diseases, mostly ischemic heart disease (Schrieks *et al*., 2013; Haseeb *et al*., 2017).

As discussed previously, prospective studies suffer from some inherent methodological flaws that may hamper the interpretation of beverage-specific health effects including cultural and behavioural aspects. In most nutrition intervention studies the effects of such confounding factor are minimized. Also, in diet-controlled nutrition intervention studies another important confounding factor, viz. the diet, is controlled for.

In intervention studies using biomarkers but not endpoints, no difference was observed when a person would consume either an equal dosage of wine, beer or spirits for a given short period of time (Van Der Gaag *et al*., 2001). This suggests that the ethanol in alcoholic beverages is the main factor contributing to metabolic changes that may explain how the consumption of these beverages (in moderation) may be associated with a reduced risk for cardiovascular diseases.

The four studies on the association between diabetes mellitus type II incidence and moderate consumption of wine, beer and spirits showed in general small effects for beer (Huang *et al*., 2017), wine (Holst *et al*., 2017) and spirits (Cullmann *et al*., 2012) each, but no consistent overall risk reduction for all three beverages. This is surprising since diabetes mellitus type II incidence has been reported to be associated with reduced risk when consuming alcohol in moderation (Koppes *et al*., 2005). The studies meeting our selection criteria were generally small and concerned populations that consumed little alcohol: the earlier reviews reported risk reductions of ~20–30% at consumptions up to 24–48 g alcohol per day.

The limitations of our study are as follows: (a) the studies varied considerably in population size, location, alcohol consumption categories, alcohol consumption registration methodology, expression of risks and control groups; (b) none of the selected studies on moderate alcohol consumption were controlled for binge drinking nor for drinking pattern (frequency of drinking, e.g. daily, weekly etc.), since binge drinking may have adversely affected outcomes; (c) not all relevant endpoints were included: several cancers clearly associated with alcohol consumption were not selected, either because no additional studies were published or because studies did not meet our selection criteria; (d) endpoints varied and included both incidence and mortality; (e) publication bias is an inevitable problem in a review of published studies; and (f) data overlap: two cohort studies on diabetes mellitus type II (Cullmann *et al*., 2012; Marques-Vidal *et al*., 2015) and one on gastric cancer (Duell *et al*., 2011) were also included in the MAs on the same topics (Huang *et al*., 2017; Wang *et al*., 2018). However, this review provided an overview of the major commonly occurring diseases and their association with light to moderate consumption of the three main alcoholic beverages.

We have evaluated the epidemiological studies published over the last ten years comparing light to moderate consumption of wine, beer and distilled spirits in their risk for main health outcomes such as all-cause mortality, various types of cancer, cardiovascular disease and diabetes type II. Based on these results, we conclude that moderate consumption of one specific alcoholic beverage (wine, beer or spirits) may not be consistently associated with higher or lower risks for these common health outcomes as compared with moderate consumption of any of the other alcoholic beverages. The current lack of differentiation between the various main alcoholic beverages in legal consequences may not need to change, also because a lack of differentiation in major health outcomes seems not to exist.

## Conflict of interest statement

Dr R. Estruch reports grants from Government of Spain (FIS, ISCIII and CIBER OBN), Cerveza y Salud and Fundación Dieta Mediterranea, Spain. Also, personal fees for given lectures from Brewers of Europe, Belgium; Fundación Cerveza y Salud, Spain; Pernaud-Ricard, Mexico, Instituto Cervantes, Alburquerque, USA; Instituto Cervantes, Milan, Italy; Instituto Cervantes, Tokyo, Japan; Lilly Laboratories, Spain and Wine and Culinary International Forum, Spain and non-financial support to organize a National Congress on Nutrition. Also feeding trials with products from Grand Fountain and Uriach Laboratories, Spain.

Dr H. Hendriks reports grants from the Government of The Netherlands (OostNL, ZonMW). Also, personal fees for lectures from Brewers of Europe, Belgium; Fundación Cerveza y Salud, Spain; CNRIFFI China; Dutch Oenologists Academy, The Netherlands; Brewers of Europe, Brussels. Paid consultancies from Carlsberg and IARD and an educational grant from Diageo plc.

## Funding

This work was supported by an unrestricted educational grant (No: 4800295861) from Diageo plc. The funder had no role in the design of the study nor in the collection, analysis or interpretation of the data.

## Supplementary Material

supplementary_agab082
