# Supplementary material for: Associations between Low to Moderate Consumption of Alcoholic Beverage Types and Health Outcomes: A Systematic Review
Source: Alcohol Alcohol. 2021 Dec 11;57(2):176–84. doi: 10.1093/alcalc/agab082 (PMC8919407; doi:10.1093/alcalc/agab082)
Supplement: supplementary_agab082 [file supplementary_agab082.docx]

**Supplementary material**

Search strategy

PubMed

(("wine"[Title/Abstract] OR "wines"[Title/Abstract]) AND ("beer"[Title/Abstract] OR "beers"[Title/Abstract])) AND ("spirits"[Title/Abstract] OR "liquor"[Title/Abstract]). Filters: Humans, English, from 2011 – 2020.

Embase

((wine:ab,ti OR wines:ab,ti) AND (beer:ab,ti OR beers:ab,ti) AND (spirit:ab,ti OR spirits:ab,ti OR liquor:ab,ti)) AND (((wine:ab,ti OR wines:ab,ti) AND (beer:ab,ti OR beers:ab,ti) AND (spirit:ab,ti OR spirits:ab,ti OR liquor:ab,ti)) AND (2011:py OR 2012:py OR 2013:py OR 2014:py OR 2015:py OR 2016:py OR 2017:py OR 2018:py OR 2019:py OR 2020:py) AND 'human'/de).

Web of Science

((wine OR wines) AND (beer OR beers) AND (spirit OR spirits OR liquor))

Refined by: LANGUAGES=(ENGLISH), 2011–2020, AND [excluding] DOCUMENT TYPES=(REVIEW) AND [excluding] DOCUMENT TYPES=(BOOK CHAPTER OR EDITORIAL MATERIAL OR MEETING ABSTRACT OR REPRINT).
